# Age at birth of first child and coronary heart disease risk factors at age 53 years in men and women: British birth cohort study

**DOI:** 10.1136/jech.2008.076943

**Published:** 2009-01-06

**Authors:** R Hardy, D A Lawlor, S Black, G D Mishra, D Kuh

**Affiliations:** 1 Medical Research Council Unit for Lifelong Health and Ageing, London, UK; 2 Medical Research Council Centre for Causal Analysis in Translational Epidemiology, Department of Social Medicine, University of Bristol, Bristol, UK

## Abstract

**Objective:**

To assess the associations between parental age at birth of first child and coronary heart disease (CHD) risk factors in men and women. To investigate whether the associations are explained by childhood predictors of age at parenthood or adult lifestyle factors related to child rearing.

**Methods:**

Data from 2540 men and women, with CHD risk factors measured at age 53 years, from a birth cohort study of individuals born in Britain in 1946 (Medical Research Council National Survey of Health and Development) and followed up regularly throughout life, were analysed.

**Results:**

Younger age at birth of first child in both men and women was associated with poorer mean body mass index (BMI), waist–hip ratio, blood pressure (BP), high-density lipoprotein cholesterol, triglyceride and glycated haemoglobin levels. Mean BMI decreased from 28.0 kg/m^2^ (95% CI 27.2 to 28.8) in the teenage motherhood group to 26.8 kg/m^2^ (25.9 to 27.7) in the oldest motherhood group (⩾30 years). For men, the equivalent mean values were 28.5 kg/m^2^ (27.3 to 29.8) and 27.1 kg/m^2^ (26.7 to 27.6). Associations with adiposity, lipid measures and glycated haemoglobin were largely explained by childhood antecedents and adult social and lifestyle variables. Associations with BP remained robust to adjustment: systolic blood pressure remained highest in teenage parents (7.5 mmHg (1.0 to 13.9) difference in women and 8.6 mmHg (0.4 to 16.8) difference in men between the youngest and the oldest parenthood groups)

**Conclusions:**

Lifestyle factors, rather than the biological impact of pregnancy, explain the relationship between age at motherhood and CHD risk factors. Family-based lifestyle interventions targeted at young parents may improve their future CHD risk.

Detrimental changes to the coronary heart disease (CHD) risk profile occur during pregnancy. The changes seen in normal pregnancy, which are necessary for fetal growth and development, include a relative degree of insulin resistance, hypertriglyceridaemia, an increase in coagulation factors, an upregulation of the inflammatory cascade and a large expansion in plasma volume.^1^^–^6 These changes are likely to be hormonally driven through acquisition of fat in early pregnancy and its rapid mobilisation later; most of these changes become manifest during the third trimester of pregnancy.^7^ If such changes persist beyond the end of the pregnancy, as has been suggested,^7^ one might expect that early motherhood would be a risk factor for CHD because any changes that take place do so at a younger age. Alternatively those who start their families later in life may be at an increased risk of CHD as older mothers are more likely to develop gestational diabetes^8^ and gestational diabetes has been found to be associated with later type 2 diabetes and cardiovascular disease.^9^ Gestational diabetes and type 2 diabetes share a similar pathophysiology. Initial insulin resistance (resulting from the pregnancy hormones in the case of gestational diabetes) develops into gestational or type 2 diabetes when pancreatic β-cell function is no longer able to maintain sufficient levels of insulin to counter the effects of the insulin resistance. It remains unclear whether the association of gestational diabetes with type 2 diabetes simply reflects this shared underlying pathophysiology, whether pregnancy unmasks an already existing hereditary or acquired risk for type 2 diabetes in the form of gestational diabetes,^7^ or whether the pregnancy (for example, as a result of response to fetal genotype^10^) causes maternal pancreatic β-cell dysfunction and gestational diabetes, which then directly increases the risk of later type 2 diabetes. Thus, biological changes related to pregnancy could plausibly increase the risk of later type 2 diabetes and CHD.

A small number of studies have examined the association of age at first child and CHD risk in women, with some finding increased risk in those with earlier age at first birth,^11^^–^14 one increased risk in those with later age^15^ and one finding no effect of age at first birth on later CHD risk.^16^ However, as with the number of children,^17^^–^19 any association of age at first birth with later CHD risk may represent behavioural differences associated with child rearing at different ages. Selection into early childbearing may also mean that those who have children at an early age have prior risk factors that are related to greater CHD, such as poor socioeconomic circumstances in childhood.^20^^–^22 These lifestyle factors and predictors of early parenthood may also influence CHD risk in men. To our knowledge no study has compared the effect of age at first child on CHD or on its risk factors in men and women as a means of distinguishing a biological from a lifestyle pathway. Further, few of the previous studies in women have adequately adjusted for potential confounding and mediating factors.

Using data on both men and women from the Medical Research Council (MRC) National Survey of Health and Development (NSHD) we have previously suggested that the relatively weak relationships between the number of children and CHD risk factors at age 53 years were more behavioural than biological.^19^ We now investigate the relationship between parental age at the birth of the first child and CHD risk factors measured at 53 years in both men and women from this cohort. We assess whether any relationships can be accounted for by factors that might predict early parenthood and then whether they are explained by the total number of children or by social class and behavioural risk factors in adult life.

## METHODS

The MRC NSHD is a birth cohort study consisting of a socially stratified sample of 2547 women and 2815 men born in one week in March 1946. There have been 21 follow-ups of the whole cohort, with the most recent being at age 53 years, when 3035 cohort members (1472 men, 1563 women) provided information. The majority (n = 2989) were interviewed and examined in their own homes by research nurses, with others completing a postal questionnaire (n = 46). Contact was not attempted for the 1979 individuals who had previously refused to take part, were living abroad, were untraced since last contact at 43 years or had already died. The responding sample at age 53 is in most respects representative of the national population of a similar age.^23^ There was greater drop out by 53 years among those from a manual social class in childhood and of low socioeconomic status in early adulthood.^23^ The data collection at age 53 years received multicentre research ethics committee approval, and informed consent was given by respondents to each set of questions and measures.

At age 53 years, height, weight and waist and hip circumference were measured using standard protocols. Body mass index (BMI), defined as weight/height^2^, and waist-to-hip ratio were calculated. Blood pressure was measured while the survey member was seated and after 5 min of rest, using the validated Omron HEM-705 (Omron, Tokyo, Japan) automated digital oscillometric sphygmomanometer. Non-fasting venous blood samples were taken. Total cholesterol, high-density lipoprotein (HDL) cholesterol, triglycerides and glycated haemoglobin (HbA1c) were measured as has been previously reported.^19^ ^24^ Low-density lipoprotein (LDL) cholesterol was calculated using the Friedewald formula. The interview nurses recorded information on participants’ current medication, including antidiabetic medication, lipid-lowering medication and medicines for high blood pressure. Dates of all live births have been collected throughout the adult life of the cohort. Age at first birth was grouped into <20 years, 20–25 years, 25–29 years, ⩾30 years.

### Childhood and adolescent factors

Potential antecedent variables used information collected at contacts up to the age of 15 years and were chosen based on previous analyses of predictors of early parenthood, particularly motherhood, in this cohort^20^ ^22^ and in the 1958 British birth cohort.^21^ Social class at age 4 years was based on the occupation of the father in 1950 and categorised into manual or non-manual. Cognitive scores at age 8 years, from verbal and non-verbal intelligence tests devised by the National Foundation for Educational Research, were used as an early measure of educational achievement. Early age at puberty is related to early initiation of sexual activity^25^ and, in the NSHD, to high BMI and, in men, high blood pressure.^26^ Boys were classified as infantile, early puberty, advanced puberty and fully mature based on observations made by a school doctor at medical examinations when the cohort were aged 15 years.^26^ Age at menarche for girls was obtained from mothers’ reports at the examination. As girls mature earlier than boys (by age 15 years over 90% of the girls had reached menarche, whereas 55% of the boys were fully mature or had reached advanced puberty) and in order to make the age at puberty variables for each sex comparable, four age at puberty groups were derived for women. Cut points were selected (⩽12 years 3 months, 12 years 4 months to 13 years 4 months, 13 years 5 months to 14 years 6 months, ⩾14 years 7 months) so that the proportion in each group was approximately the same as for the men. Those who had not reached menarche by the age of 15 years were included in the latest group.

### Adult social and behavioural factors

Potential adult mediating variables were selected on the basis that they may be influenced by age at parenthood while also being risk factors for cardiovascular disease. Social class (non manual, manual) based on the cohort member’s own current or most recent occupation reported at age 53 years was used as an indicator of adult socioeconomic position. Physical activity at 53 years assessed by questionnaire was divided into active (done any physical activity in the last 4 weeks) and inactive and self-reported current cigarette smoking status (smoker, non-smoker) was defined. Highest educational qualification achieved (no qualifications, below O level, O level or equivalent, A level and above) was considered as an adult factor because although education is a potential predictor of age at parenthood, early parenthood would also influence the level of qualification obtained. For women, menopausal status (premenopause/perimenopause, postmenopause, hysterectomy, hormone replacement therapy (HRT)) was defined based on self-reported menstrual characteristics and self-reports of hysterectomy operations and use of HRT collected annually in postal questionnaires.^27^

### Statistical methods

Normal regression models were fitted for BMI and waist-to-hip ratio. To account for the fact that some individuals were taking medication for hypertension (n = 364), high cholesterol (n = 62) or diabetes (n = 40) at the time the CHD risk factor measurements were taken, censored normal regression models were fitted for systolic blood pressure (SBP) and diastolic blood pressure (DBP), lipids and HbA1c. These models censor the outcome measure of individuals on the associated medication at the observed value, so that the true value is assumed to be at least as high as that observed on treatment.^28^ Triglycerides and glycated haemoglobin had skewed distributions and were thus log_e_ transformed and, for the regression analyses, multiplied by 100. Regression coefficients thus represent a percentage change in outcome per unit change in exposure.^29^ For each CHD risk factor tests for trend across the four ages at first birth groups were carried out and the regression coefficient for a 1-year increase in age at first birth was estimated. In the sample with complete information on all variables (n between 1484 and 1819 depending on outcome), unadjusted results were compared with those adjusted for childhood and adolescent confounding factors using multivariable regression models. Adjustment was then made for the number of children and then additionally for the mediating adult social and behavioural variables. Analyses were carried out separately for men and women, and a final adjustment for menopausal status was carried out in women. Tests for interaction between sex and age at birth of first child were performed to assess whether a difference in association existed between the sexes both before and after adjustment. Analyses were conducted using STATA.^30^ To assess the impact of missing covariate values, analyses were repeated on imputed data sets. SAS release 9.1^31^ was used to impute 100 datasets for each analysis using PROC MI with the method of Markov chain Monte Carlo.

## RESULTS

Of the 2989 study members visited by a nurse at 53 years, 430 had no children. A further 19 were excluded because of missing data on all outcomes or age at birth of first child, leaving a total of 2540 study members for analysis. A greater proportion of excluded men (7% vs 3% included) and women (20% vs 14% included) had their first child as a teenager. Descriptive statistics for the CHD risk factors are presented in table 1. The distribution of age at birth of first child was different for men and women. More women than men became a parent under the age of 20. The range of age at first birth was 15–43 years for women and 16–51 years for men.

**Table 1:** Characteristics of men and women aged 53 years

Variable	Women		Men	
	n (%)	Mean (SD)	n (%)	Mean (SD)
BMI at 53 years (kg/m^2^)	1311	27.5 (5.4)	1206	27.5 (4.0)
Waist–hip ratio at 53 years (%)	1318	80.7 (6.6)	1205	93.7 (6.1)
Systolic blood pressure at 53 years (mmHg)	1299	133 (20)	1202	140 (20)
Diastolic blood pressure at 53 years (mmHg)	1299	82 (11)	1202	87 (12)
Total cholesterol at 53 years (mmol/l)	1134	6.12 (1.08)	1067	6.06 (1.07)
LDL cholesterol at 53 years (mmol/l)	1089	3.50 (0.99)	945	3.57 (0.93)
HDL cholesterol at 53 years (mmol/l)	1092	1.83 (0.48)	951	1.48 (0.41)
Triglycerides at 53 years (mmol/l)*	1133	1.54 (0.81)	1065	2.08 (1.21)
Glycated haemoglobin at 53 years (%)*	1140	5.62 (0.58)	1076	5.62 (0.54)
**Age at birth of first child**				
Under 20 years	181 (13.6)		40 (3.3)	
20–24 years	608 (45.8)		385 (31.8)	
25–29 years	396 (29.8)		509 (42.0)	
30 years and over	143 (10.8)		278 (22.9)	

*Geometric mean and SD calculated as described by Cole.^29^

BMI, body mass index; HDL, high-density lipoprotein; LDL, low-density lipoprotein.

As only minor differences in regression coefficients were obtained from the imputed dataset analyses when compared with the complete case analyses, only results from the latter are presented.

There was a consistent trend towards worse risk factor levels at age 53 years with younger parental age at first birth for both men and women (table 2). Only total and LDL cholesterol showed no trend with age at first child in either sex. There were no significant differences in trend between men and women according to the tests for sex interaction (p⩾0.2 in all cases) (table 2). Similar associations were found when age at first birth was considered as a continuous variable (table 2).

**Table 2:** Means of coronary heart disease (CHD) risk factors at 53 years by parental age at birth of first child and regression coefficients representing mean change in risk factor per year increase in parental age at birth of first child

Outcome	N	Age at birth of first child				p Value		Age (years) at birth of first child as a continuous variable	
		<20 years	20–24 years	25–29 years	⩾30 years				
		Mean (95% CI)	Mean (95% CI)	Mean (95% CI)	Mean (95% CI)	Trend	Sex int.*	Regression coefficient† (95% CI)	p Value
BMI (kg/m^2^)							0.5		
Women	1311	28.0 (27.2 to 28.8)	27.8 (27.3 to 28.2)	27.1 (26.5 to 27.6)	26.8 (25.9 to 27.7)	0.006		−0.08 (−0.15 to −0.01)	0.02
Men	1206	28.5 (27.3 to 29.8)	28.2 (27.8 to 28.6)	27.2 (26.9 to 27.6)	27.1 (26.7 to 27.6)	<0.001		−0.08 (−0.13 to −0.04)	<0.001
Waist–hip ratio (%)							0.9		
Women	1318	81.8 (80.8 to 82.7)	80.7 (80.2 to 81.3)	80.2 (79.6 to 80.8)	80.4 (79.4 to 81.5)	0.02		−0.09 (−0.18 to −0.01)	0.02
Men	1205	95.1 (93.2 to 97.0)	94.6 (94.0 to 95.3)	93.2 (92.6 to 93.7)	93.1 (92.4 to 93.8)	0.001		−0.13 (−0.20 to −0.07)	<0.001
SBP (mmHg)							0.6		
Women	1299	138.5 (135.2 to 141.8)	136.6 (134.8 to 138.3)	132.8 (130.6 to 134.9)	131.0 (127.4 to 134.6)	<0.001		−0.58 (−0.86 to −0.31)	0.001
Men	1202	149.5 (142.9 to 156.1)	144.0 (141.8 to 146.1)	140.0 (138.2 to 141.9)	140.6 (138.1 to 143.2)	0.003		−0.31 (−0.54 to −0.08)	0.008
DBP (mmHg)							0.6		
Women	1299	84.9 (83.0 to 86.9)	83.7 (82.7 to 84.8)	82.2 (80.9 to 83.4)	82.0 (79.9 to 84.0)	0.006		−0.23 (−0.39 to −0.08)	0.004
Men	1202	91.4 (87.3 to 95.5)	90.3 (89.0 to 91.7)	87.5 (86.3 to 88.7)	87.7 (86.1 to 89.2)	0.002		−0.21 (−0.35 to −0.06)	0.005
TC (mmol/l)							0.5		
Women	1134	6.12 (5.94 to 6.29)	6.18 (6.08 to 6.27)	6.12 (6.00 to 6.23)	6.10 (5.91 to 6.30)	0.6		−0.001 (−0.02 to 0.01)	0.9
Men	1067	6.04 (5.68 to 6.39)	6.09 (5.97 to 6.20)	6.12 (6.02 to 6.23)	6.11 (5.97 to 6.25)	0.7		0.001 (−0.01 to 0.01)	0.8
LDL-C (mmol/l)							0.6		
Women	1089	3.56 (3.40 to 3.72)	3.54 (3.46 to 3.63)	3.49 (3.38 to 3.60)	3.43 (3.25 to 3.61)	0.2		−0.005 (−0.02 to 0.009)	0.5
Men	945	3.57 (3.25 to 3.89)	3.62 (3.52 to 3.73)	3.62 (3.53 to 3.72)	3.56 (3.44 to 3.69)	0.6		−0.003 (−0.01 to 0.009)	0.6
HDL-C (mmol/l)							0.2		
Women	1092	1.72 (1.64 to 1.79)	1.83 (1.79 to 1.87)	1.89 (1.83 to 1.94)	1.89 (1.80 to 1.98)	0.001		0.009 (0.002 to 0.02)	0.01
Men	951	1.55 (1.41 to 1.70)	1.45 (1.40 to 1.49)	1.51 (1.47 to 1.55)	1.52 (1.46 to 1.57)	0.1		0.005 (0.00 to 0.01)	0.05
Triglycerides (mmol/l)‡									
Women	1133	1.68 (1.54 to 1.82)	1.59 (1.52 to 1.66)	1.45 (1.37 to 1.53)	1.52 (1.38 to 1.67)	0.008	1.0	−0.83 (−1.6 to −0.11)	0.02
Men	1065	2.10 (1.73 to 2.55)	2.25 (2.11 to 2.40)	2.10 (1.99 to 2.22)	2.00 (1.86 to 2.16)	0.03		−1.05 (−1.7 to −0.36)	0.003
HbA1c (%)‡							0.2		
Women	1140	5.64 (5.54 to 5.73)	5.65 (5.60 to 5.70)	5.59 (5.53 to 5.66)	5.59 (5.49 to 5.70)	0.2		−0.14 (−0.28 to 0.004)	0.06
Men	1076	5.75 (5.57 to 5.93)	5.68 (5.62 to 5.74)	5.59 (5.54 to 5.64)	5.57 (5.50 to 5.64)	0.003		−0.13 (−0.24 to −0.01)	0.03

*p Value for interaction between sex and age at birth of first child.

†Regression coefficient represents mean change in CHD risk factor per year increase in parental age at birth of first child.

‡Geometric means with their 95% CIs and for regression model outcomes transformed using 100×log_e_ so that regression coefficient represents the per cent change in outcome per year of age at birth of first child.

BMI, body mass index; DBP, diastolic blood pressure; HDL-C, high-density lipoprotein cholesterol; LDL-C, low-density lipoprotein cholesterol; SBP, systolic blood pressure; TC, total cholesterol.

In the sample with complete information, unadjusted associations were generally slightly weaker than those presented in table 2 due to reduced statistical power (table 3). The BMI was 1.8 kg/m^2^ (95% CI 0.4 to 3.3) higher in women who had their first child as a teenager than in those who had their first child at age 30 years or over (table 3). The difference was very similar among men (1.8 kg/m^2^, 95% CI 0.4 to 3.2). SBP was 7.7 mmHg (95% CI 1.9 to 13.5) higher in the teenage motherhood group and 8.1 mmHg (0.1 to 16.0) higher in the teenage fatherhood group than in the latest parenthood groups. For all outcomes where an unadjusted trend with age at birth of first child was observed, adjustment for childhood social class, cognitive score at age 8 years and age at puberty attenuated the associations. This was particularly the case for BMI, waist-to-hip ratio and triglycerides among mothers. Adjustment for these confounders did not have such a marked effect on the associations with SBP or DBP.

**Table 3:** Regression coefficients for the association between parental age at birth of first child and coronary heart disease (CHD) risk factors at age 53 years: unadjusted and adjusted for childhood and adolescent factors (childhood social class, cognitive score at age 8 years, age at puberty)

Outcome	n	Unadjusted							Adjusted for childhood and adolescent factors						
		Regression coefficient and 95% confidence interval compared with baseline group of ⩾30 years							Regression coefficient and 95% CI compared with baseline group of ⩾30 years						
		<20 years		20–25 years		25–29 years		p Value trend	<20 years		20–25 years		25–29 years		p Value trend
BMI (kg/m^2^)															
Women	939	1.8	0.4 to 3.3	1.0	−0.2 to 2.1	0.2	−1.0 to 1.5	0.002	0.8	−0.6 to 2.3	0.4	−0.8 to 1.5	0.0	−1.2 to 1.2	0.7
Men	877	1.8	0.4 to 3.2	1.2	0.5 to 2.0	0.4	−0.3 to 1.1	<0.001	1.1	−0.4 to 2.5	0.8	0.1 to 1.6	0.3	−0.4 to 1.0	0.01
Waist–hip ratio (%)															
Women	942	1.3	−0.4 to 3.0	0.3	−1.1 to 1.7	−0.3	−1.8 to 1.2	0.06	0.4	−1.4 to 2.1	−0.2	−1.6 to 1.2	−0.4	−1.9 to 1.1	0.9
Men	877	2.6	0.4 to 4.8	2.0	0.9 to 3.1	0.5	−0.5 to 1.6	<0.001	1.5	−0.7 to 3.8	1.3	0.1 to 2.5	0.2	−0.8 to 1.3	0.005
SBP (mmHg)															
Women	928	7.7	1.9 to 13.5	6.3	1.6 to 10.9	2.1	−2.8 to 7.0	<0.001	6.8	0.8 to 12.8	6.0	1.3 to 10.7	2.1	−2.8 to 7.0	0.002
Men	874	8.1	0.1 to 16.0	4.4	0.3 to 8.4	−0.8	−4.6 to 3.0	0.003	5.8	−2.2 to 13.8	2.8	−1.3 to 6.9	−1.2	−5.1 to 2.6	0.04
DBP (mmHg)															
Women	928	3.0	−0.4 to 6.5	2.2	−0.6 to 4.9	0.3	−2.6 to 3.2	0.01	2.5	−1.0 to 6.0	2.0	−0.8 to 4.7	0.2	−2.7 to 3.1	0.04
Men	874	4.0	−0.9 to 8.9	3.8	1.3 to 6.3	0.5	−1.8 to 2.9	0.001	2.1	−2.8 to 7.0	2.5	−0.02 to 5.0	0.2	−2.2 to 2.5	0.04
TC (mmol/l)															
Women	815	−0.02	−0.34 to 0.29	0.17	−0.09 to 0.42	0.10	−0.17 to 0.37	0.8	−0.06	−0.39 to 0.26	0.14	−0.17 to 0.40	0.09	−0.18 to 0.36	1.0
Men	782	−0.09	−0.51 to 0.33	0.03	−0.19 to 0.25	−0.04	−0.24 to 0.16	0.9	−0.15	−0.57 to 0.28	0.01	−0.21 to 0.24	−0.04	−0.24 to 0.17	0.9
LDL-C (mmol/l)															
Women	785	0.15	−0.14 to 0.44	0.18	−0.05 to 0.42	0.12	−0.13 to 0.37	0.2	0.11	−0.19 to 0.41	0.16	−0.08 to 0.40	0.12	−0.13 to 0.37	0.4
Men	691	−0.03	−0.41 to 0.34	0.06	−0.13 to 0.26	0.06	−0.12 to 0.25	0.8	−0.05	−0.43 to 0.33	0.06	−0.14 to 0.26	0.06	−0.12 to 0.25	0.8
HDL-C (mmol/l)															
Women	787	−0.18	−0.32 to −0.04	−0.03	−0.14 to 0.09	0.00	−0.12 to 0.12	0.01	−0.14	−0.28 to 0.01	−0.01	−0.12 to 0.11	0.00	−0.17 to 0.12	0.1
Men	697	−0.02	−0.18 to 0.15	−0.07	−0.15 to 0.02	−0.04	−0.12 to 0.04	0.2	−0.02	−0.19 to 0.15	−0.06	−0.15 to 0.02	−0.03	−0.11 to 0.05	0.2
Triglycerides (%)*															
Women	815	6.9	−7.9 to 21.7	4.5	−7.4 to 16.5	−0.9	−13.6 to 11.7	0.1	1.1	−13.9 to 16.1	1.4	−10.6 to 13.4	−1.0	−13.5 to 11.6	0.7
Men	780	6.2	−16.5 to 28.8	15.8	4.1 to 27.6	4.5	−6.6 to 15.5	0.02	−0.06	−23.0 to 22.8	12.4	0.4 to 24.4	3.6	−7.5 to 14.7	0.1
HbA1c (%)*															
Women	817	2.2	−0.6 to 5.1	1.7	−0.7 to 4.0	1.5	−1.0 to 3.9	0.1	1.2	−1.7 to 4.2	1.1	−1.3 to 3.4	1.2	−1.3 to 3.7	0.5
Men	788	2.3	−1.3 to 6.0	2.7	0.8 to 4.6	0.6	−1.2 to 2.4	0.003	2.2	−1.5 to 5.9	2.6	0.6 to 4.5	0.6	−1.2 to 2.4	0.007

*Outcomes transformed using 100×log_e_ so that regression coefficients represent percent change in outcome for each group compared with baseline group.

BMI, body mass index; DBP, diastolic blood pressure; HbA1c, glycated haemoglobin; HDL-C, high-density lipoprotein cholesterol; LDL-C, low-density lipoprotein cholesterol; SBP, systolic blood pressure; TC, total cholesterol.

The addition of the total number of children to the model did not substantively alter the associations in either sex (full results available on request). After further adjustment for adult social class, cigarette smoking and physical activity at 53 years and educational qualifications, the effects of age at first birth were attenuated for glycated haemoglobin among men, and BMI and HDL cholesterol among women (table 4). A sex difference in the association for waist-to-hip ratio (p = 0.02) was observed. Among men, but not women, a significant negative relationship was still evident. Similarly for BMI, a relationship remained among men, but not women (test for sex interaction p = 0.1). In both sexes the association between age at first child and SBP remained with the youngest mothers having a mean SBP 7.5 mmHg (1.0 to 13.9) higher than the oldest. The difference was 8.6 mmHg (0.4 to 16.8) in men. Similarly, for DBP there was little attenuation after addition of these potential mediators. Adjustment for menopausal status in women had little impact on these findings.

**Table 4:** Regression coefficients for the association between parental age at birth of first child and coronary heart disease (CHD) risk factors at age 53 years, adjusted for childhood and adolescent factors and additionally for number of children and adult lifestyle and behavioural factors (educational qualifications at age 26 years, adult social class, cigarette smoking, physical activity at 53 years)

Outcome	n	Regression coefficient and 95% CIs compared with baseline group of ⩾30 years						p Value	
		<20 years		20–24 years		25–29 years		Trend	Sex int.*
BMI (kg/m^2^)									0.1
Women	939	0.0	−1.5 to 1.6	−0.1	−1.3 to 1.2	0.0	−1.2 to 1.2	0.99	
Men	877	1.1	−0.4 to 2.6	0.9	0.1 to 1.7	0.4	−0.3 to 1.1	0.01	
Waist–hip ratio (%)									0.02
Women	942	−0.7	−2.6 to 1.2	−0.8	−2.3 to 0.7	−0.5	−2.0 to 1.0	0.4	
Men	877	1.5	−0.7 to 3.8	1.3	0.2 to 2.5	0.2	−0.8 to 1.3	0.02	
SBP (mmHg)									0.8
Women	928	7.5	1.0 to 13.9	6.5	1.5 to 11.5	2.9	−2.1 to 7.9	0.004	
Men	874	8.6	0.4 to 16.8	3.7	−0.5 to 8.0	−0.8	−4.6 to 3.1	0.01	
DBP (mmHg)									0.7
Women	928	2.8	−1.0 to 6.6	2.4	−0.6 to 5.3	0.7	−2.3 to 3.7	0.05	
Men	874	3.2	−1.8 to 8.3	3.0	0.3 to 5.6	0.3	−2.1 to 2.7	0.02	
TC (mmol/l)									0.9
Women	815	−0.07	−0.42 to 0.29	0.14	−0.13 to 0.41	0.12	−0.16 to 0.39	0.9	
Men	782	−0.15	−0.59 to 0.29	−0.01	−0.24 to 0.22	−0.05	−0.25 to 0.16	0.8	
LDL-C (mmol/l)									0.9
Women	785	0.05	−0.28 to 0.37	0.13	−0.12 to 0.38	0.12	−0.13 to 0.37	0.7	
Men	691	−0.06	−0.45 to 0.34	0.06	−0.15, 0.27	0.05	−0.13 to 0.24	0.8	
HDL-C (mmol/l)									0.6
Women	787	−0.07	−0.23 to 0.08	0.02	−0.09 to 0.14	0.01	−0.12 to 0.13	0.6	
Men	697	−0.02	−0.20 to 0.15	−0.07	−0.16 to 0.02	−0.03	−0.11 to 0.05	0.2	
Triglycerides (%)†									0.2
Women	815	−3.6	−19.8 to 12.6	−1.6	−14.1 to 10.9	−1.3	−14.0 to 11.4	0.7	
Men	780	−0.3	−23.9 to 23.2	11.8	−0.6 to 24.2	3.7	−7.4 to 14.9	0.1	
HbA1c (%)†									0.2
Women	817	−0.1	−3.3 to 3.0	0.5	−2.0 to 2.9	1.4	−1.1 to 3.9	0.6	
Men	788	1.0	−2.7 to 4.8	1.5	−0.4 to 3.5	0.3	−1.5 to 2.1	0.1	

*p Value for interaction between sex and age at birth of first child.

†Outcomes transformed using 100×log_e_ so that regression coefficients represent percent change in outcome for each group compared with baseline group.

BMI, body mass index; HbA1c, glycated haemoglobin; HDL-C, high-density lipoprotein cholesterol; LDL-C, low-density lipoprotein cholesterol; SBP, systolic blood pressure; TC, total cholesterol.

## DISCUSSION

Younger parental age at birth of first child was consistently related with poorer CHD risk factor levels in both sexes. The associations between age at first becoming a parent and measures of adiposity, adverse lipid profiles and glycated haemoglobin were explained by a combination of childhood and adolescent confounding factors and mediation by adult social and behavioural factors, particularly among women. Associations with blood pressure remained robust to adjustment.

### Strengths and limitations

Our research advances previous studies by presenting associations for men and women, which enables us to explore the extent to which any associations are likely to be driven by pregnancy-related factors. It also provides information on the impact of age at fatherhood on CHD risk. Our study has the advantage that the age at which children were born was reported in the same way for men and women, and was collected prospectively over the reproductive years rather than being recalled in later life. It was also possible to adjust for a range of prospectively collected lifetime confounding and mediating variables.

Our assays were undertaken on non-fasting samples. Glycated haemoglobin is an accurate measure of mean blood glucose concentration over the half-life of a red blood cell (usually 60 days) that does not require fasting to be accurate and reliable. Total cholesterol and HDL cholesterol assessed on non-fasting samples are also reliable.^32^ Although non-fasting triglyceride levels may differ from fasting levels, they have been shown to be strongly and independently related to cardiovascular disease events.^33^ ^34^ We were restricted to looking at the associations with CHD risk factors, rather than CHD morbidity or mortality, due to lack of numbers with disease outcomes and we do not know whether the relationships seen here would also be seen for CHD mortality. Those most at risk will have already died from CHD by the age 53 year follow-up and if they also tended to have their first child at a young age the observed associations would be weaker than the true associations. Due to missing confounding and mediating data, the sample size in multivariable analyses was reduced. Sensitivity analyses using multiple imputations to allow for missing covariate values, showed our conclusions from complete case analyses to be robust. The censored normal regression models used for dealing with individuals on treatment make the assumption that the distribution of the underlying measurement above any given threshold in treated individuals is the same as the corresponding distribution of observed measurements in the untreated group. However, this method has been found to be robust to violations of this assumption.^35^

### Comparison with other studies

Several studies have considered age at birth of first child and heart disease in women only. No other studies have compared men and women, and few have adjusted for the range of potential confounders and mediators available in our study. Consistent with our findings, a large population-based cohort study from Sweden found increased risk of premature death from ischaemic heart disease in teenage mothers^14^ compared with those who became mothers aged 20–29 years. This was after adjustment for socioeconomic characteristics after childbirth, but not behavioural or lifestyle characteristics or pre-childbirth factors. Three US case–control studies have observed increased risks of myocardial infarction,^12^ CHD^13^ and sudden cardiac death^11^ among women who had been teenage mothers. The increased risk of sudden cardiac death was accounted for by cigarette smoking.^11^ In contrast, a large US cohort study of nurses found no significant association between age at first birth and CHD,^16^ whereas another smaller cohort found that women who had their first child later in life had an increased risk of heart disease (age-adjusted RR 2.90, 95% CI 1.21–6.93) for ages 33–43 compared with 25–29).^15^ Research using National Health and Nutrition Examination Survey (NHANES) III data, and like our study considering CHD risk factors, found delayed childbearing (having any child born when mother was over 35 years) to be associated with increased odds of high blood pressure and glucose abnormalities, but not abnormal lipid levels.^36^ However, this study is not directly comparable with ours as it did not investigate early motherhood and it considers the birth of any child rather than first child.

### Possible explanations and implications

The associations with age at becoming a parent were stronger and more consistent than those previously observed in the same study with number of children.^19^ Most of the observed relationships were attenuated by a combination of confounding by early life socioeconomic position, cognitive score and age at puberty or mediation by adult socioeconomic position and health related behaviours. Early parenthood was related to lower childhood and adult socioeconomic status, lower educational qualifications and, particularly in women, lower levels of activity and higher levels of cigarette smoking. This suggests lifelong lower socioeconomic status, lower educational attainment and subsequent poorer lifetime health behaviours^37^ may be the pathway by which early parenthood is related to poorer CHD risk factors. Although associations were observed in both sexes, more women than men became parents under the age of 25 years meaning that a greater number of women were at increased CHD risk as a result of this pathway from early parenthood. That some associations, in particular that with the waist-to-hip ratio, appeared to be stronger in men than women after adjustment suggested that there may be confounding variables specific to men that have not been identified. This may be because less research exists on the predictors and consequences of early fatherhood than early motherhood.^38^ ^39^

There was less confounding or mediation of the relationship with blood pressure, particularly SBP. Similar associations in both men and women rules out a pregnancy related explanation. It might be that becoming a parent at an early age results in increased stress, which via the autonomic nervous system, results in permanent increases in blood pressure in both sexes. Further studies would be required to test this hypothesis.

Future research to investigate in more detail the social pathways linking early parenthood with higher CHD risk is needed in younger cohorts of men as well as women. The potential of family-based lifestyle interventions targeted at young mothers and fathers as a way to improve future CHD risk requires investigation.

**What this study adds**

- This study shows that the relationships between younger age at first becoming a parent and poorer CVD risk factors at age 53 years are very similar for fathers as well as mothers.
- The attenuation of the relationships after adjustment for potential predictors of early parenthood and adult mediators suggest that social and lifestyle-related factors drive the associations.

**Policy implications**

Family based interventions to promote a healthier lifestyle targeted at young mothers and fathers may be a way to improve future CHD risk.
